# Dynamic interplay of ParA with the polarity protein, Scy, coordinates the growth with chromosome segregation in *Streptomyces coelicolor*

**DOI:** 10.1098/rsob.130006

**Published:** 2013-03

**Authors:** Bartosz Ditkowski, Neil Holmes, Joanna Rydzak, Magdalena Donczew, Martyna Bezulska, Katarzyna Ginda, Paweł Kędzierski, Jolanta Zakrzewska-Czerwińska, Gabriella H. Kelemen, Dagmara Jakimowicz

**Affiliations:** 1 Ludwik Hirszfeld Institute of Immunology and Experimental Therapy, Polish Academy of Sciences, Wroclaw, Poland; 2 University of East Anglia, Norwich Research Park, Norwich, UK; 3 Faculty of Biotechnology, University of Wroclaw, Wroclaw, Poland; 4 Technical University of Wroclaw, Wroclaw, Poland

**Keywords:** chromosome segregation, ParA, polar growth, bacterial development, sporulation

## Abstract

*Prior to* bacterial cell division, the ATP-dependent polymerization of the cytoskeletal protein, ParA, positions the newly replicated origin-proximal region of the chromosome by interacting with ParB complexes assembled on *parS* sites located close to the origin. During the formation of unigenomic spores from multi-genomic aerial hyphae compartments of *Streptomyces coelicolor*, ParA is developmentally triggered to form filaments along the hyphae; this promotes the accurate and synchronized segregation of tens of chromosomes into prespore compartments. Here, we show that in addition to being a segregation protein, ParA also interacts with the polarity protein, Scy, which is a component of the tip-organizing centre that controls tip growth. Scy recruits ParA to the hyphal tips and regulates ParA polymerization. These results are supported by the phenotype of a strain with a mutant form of ParA that uncouples ParA polymerization from Scy. We suggest that the ParA–Scy interaction coordinates the transition from hyphal elongation to sporulation.

## 2. Introduction

In bacteria, the faithful distribution of chromosomes and low-copy plasmids into daughter cells is achieved by an active partitioning system [1]. In rod-shaped cells, the chromosomal partitioning system drives the rapid movement of chromosomal origin regions (*oriC*) towards the cell pole(s) soon after their replication. In most bacteria, there are three core components of the partitioning machinery: ParA, a cytoskeletal Walker A type ATPase that provides the force for segregation; ParB, a DNA-binding protein that organizes the segregation of nucleoprotein complexes; and origin-proximal ParB-binding DNA sequences called *parS* [2–4]. Structural and biochemical studies of a ParA homologue from *Thermus thermophilus* showed that it forms a sandwich dimer and polymerizes into filaments in the presence of ATP and DNA [5]. The ability of ParA to form ATP-dependent polymers is reflected by its dynamic localization in the cell. Pole-to-pole oscillation has been observed *in vivo* for plasmid ParA homologues [6], and some chromosomal ParAs reportedly localize at the cell pole(s) and form filamentous structures that extend towards the opposite poles [7–9]. It has been suggested that the shrinking of the ParA structure towards the pole is associated with the translocation of ParB complexes, which disassemble the ParA filament by providing a ‘pulling’ mechanism [1].

Although these recent studies have provided new insight into the dynamics of ParA filaments, many other issues have yet to be addressed, including their architecture, the molecular mechanisms by which the filaments form and generate chromosomal movement, and the involvement of other cellular components in ParA-mediated segregation. ParA proteins from various bacteria share some features, such as polymerization and ParB-stimulated ATPase activity, but they also vary in some respects. For example, the polymerization and dynamic localization of ParA usually require its ATPase activity, but the influences of DNA and ParB on ParA polymerization can vary across bacteria [1].

Studies of chromosome segregation in the mycelial genus, *Streptomyces*, have provided additional insights into the properties of ParA proteins. In a complex life cycle reminiscent of filamentous fungi, the growth of *Streptomyces coelicolor* colonies starts with the formation of branched vegetative hyphae, proceeds through the emergence of reproductive aerial hyphae, which are finally converted into long chains of spores. In the vegetative mycelium, septa occasionally separate adjacent multi-genomic compartments, but there is no obvious chromosome segregation. The hyphae elongate by tip extension, which depends on a number of self-assembling coiled-coil proteins, including (i) the polarity determinant, DivIVA, (ii) the long coiled-coil protein, Scy, and (iii) the filamentous FilP [10–13]. Changes in the levels of these proteins visibly affect hyphal shape and/or tip extension and branching. The observed interactions among these three proteins suggest that polarized growth in *Streptomyces* is controlled by a dedicated polarisome-like assembly, named the ‘tip-organizing centre’ (TIPOC) [14,15].

During tip elongation, replicating chromosomes follow the growing tip [16]. Recent work has suggested that the tip-associated DivIVA may interact with the chromosome segregation apparatus in Actinomycetes [17]. Rapid aerial hyphae growth accompanied by intensive chromosome replication leads to the formation of an elongated sporogenic compartment containing tens of non-segregated chromosomes. Conversion of these compartments into chains of unigenomic spores requires the cessation of growth and chromosome replication, followed by synchronous chromosome segregation accompanied by the regular placement of FtsZ-rings along the compartment [18,19]. In contrast to the movement of chromosomes towards the pole(s) of rod-shaped bacteria, tens of *Streptomyces* chromosomes are condensed and uniformly arranged along the hyphal tip compartment, ensuring that each prespore receives only a single copy of the chromosome [20].

Despite the fact that the components of the *Streptomyces* partitioning system share many biochemical properties with their counterparts in rod-shaped unicellular bacteria, there are some rather remarkable differences. For example, *Streptomyces* ParA has a complex localization pattern that depends on the growth stage; it is present at the tips of vegetative and young aerial hyphae, but during further aerial growth it extends along the apical compartments [21]. Meanwhile, ParB binds to numerous *parS* sites near *oriC* to form large complexes that are arrayed regularly along the aerial hyphae tip compartment and disassemble soon after the completion of septation [20,22]. ParA mediates the efficient assembly of ParB complexes *in vivo* and *in vitro*, and both proteins are important for the proper distribution of chromosomes in aerial hyphae before septation. Deletion of *parA* or *parB* results in frequent anucleate spores (about 24% and 15%, respectively), and elimination of ParA also strongly affects sporulation septation. We previously postulated that ParA provides scaffolding for the proper distribution of ParB complexes and controls the synchronized segregation of multiple chromosomes, possibly by mediating a segregation and septation checkpoint [21,23].

Interestingly, FtsZ localization and DNA distribution in spores were altered in a *scy* mutant, suggesting that cell division and chromosome segregation during sporulation require a functional TIPOC [14]. Observations that ParA also localizes to the hyphal tips raised the question of whether ParA is part of TIPOC and whether there is a direct link between cell division and polarized growth in *Streptomyces*. Similar to its orthologues in other bacteria, *Streptomyces* ParA shows ATP-dependent polymerization *in vitro* [24]. However, we showed that *Streptomyces* ParA polymerization is unusual in that it is negatively regulated by a novel Actinobacteria-specific interaction partner, ParJ [24]. ParJ and the recently described TipN in *Caulobacter crescentus* [7,8] are the first proteins other than ParB that reportedly regulate ParA filamentation. Here, we show for the first time that there is a direct molecular link between chromosome segregation and polarized growth in *Streptomyces*. We further show that Scy controls *Streptomyces* ParA polymerization, and demonstrate that a dynamic interplay between Scy and ParA at the aerial hyphal tip triggers the developmental switch from hyphal extension to sporulation.

## 3. Results

### 3.1. There is a direct interaction between ParA and the tip-organizing centre protein, Scy

**Figure 1.**
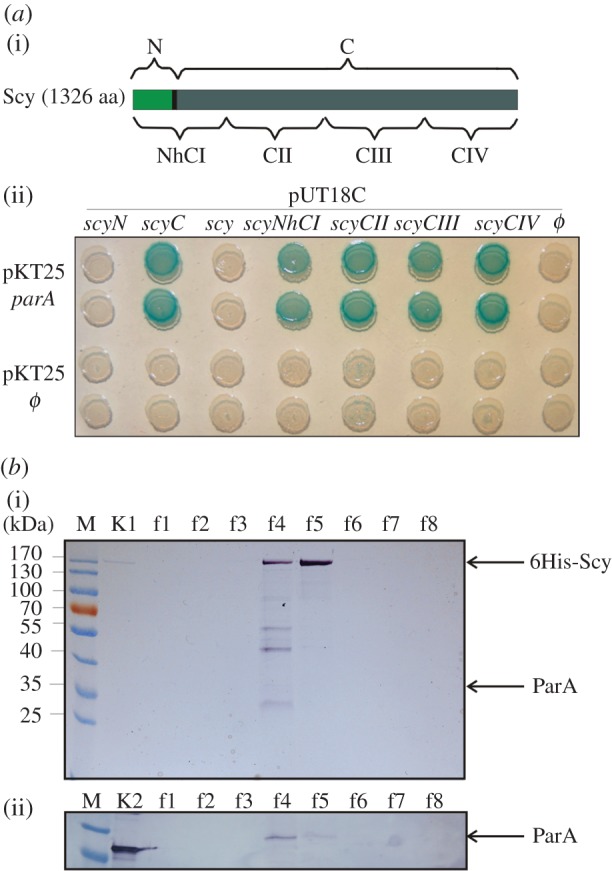
Interaction of ParA with Scy. (*a*) Interaction of ParA with Scy in the bacterial two-hybrid (BTH) system. (i) Scheme of the Scy protein and the constructs used in this study. (ii) Interaction of T25-ParA with T18-fused fragments of Scy. Blue colony colour indicates an interaction of the analysed proteins. (*b*) Co-purification of 6His-Scy/ParA complexes from *S. coelicolor*. Recombinant 6His-Scy was purified from an extract of thiostrepton-induced strain M145pK48 using metal-ion affinity chromatography. The presence of ParA in the Scy elution fractions was detected with an anti-ParA antibody. (i) SDS-PAGE of Scy purification fractions. Lanes: M, molecular weight marker; K1, purified 6His-Scy protein; f1–f8, imidazole elution fractions. (ii) Western blotting with an anti-ParA antibody. Lanes: as for (i), except K2, purified ParA protein.

The dynamic, developmentally regulated tip localization of *S. coelicolor* ParA prompted us to search for additional ParA-interacting partners using a bacterial two-hybrid (BTH), which is based on the activation of a *lacZ* gene expression when the two halves of a split adenylate cyclase are brought together by the interaction of their fusion partners [25]. In the BTH library, in addition to ParJ [24], we identified a clone encoding a 348 amino acid (aa)-long C-terminal part (978 aa–1326 aa) of Scy, a novel large coiled-coil protein responsible for polarized growth [14]. Sequence analysis suggested that Scy is composed of two domains separated by a hinge region: a short (63 aa) N-terminal canonical coiled-coil domain; and a very long (1226 aa) C-terminal domain dominated by a unique non-canonical coiled-coil [12]. The interaction of ParA and Scy *in vivo* was confirmed using the BTH system. ParA and Scy (whole and truncated) were fused to the adenylate cyclase fragments, and their ability to interact was assayed (see the electronic supplementary material, table S1; figure 1). An N-terminally truncated Scy protein encompassing only the non-canonical coiled-coil domain (T18C-ScyC; 66–1326 aa) gave a positive signal with ParA, whereas the N-terminal Scy domain containing the hinge region (T18C-ScyN; 1–73 aa) and full-length Scy (T18C-Scy) failed to interact with ParA (figure 1). To determine if the interaction interface extends beyond the originally identified C-terminal fragment of Scy (348 aa), we divided the long C-terminal domain of Scy into three shorter fragments of about 350 aa each (figure 1). Surprisingly, the BTH assay showed that each of the analysed fragments (ScyCII, ScyCIII and ScyIV) was bound by ParA. We further examined whether the N-terminal domain might actively interfere with the BTH interaction, as suggested by the negative result obtained with the full-length T18-Scy fusion. The T18-ScyNhCI fusion protein, which contained the N-terminal domain, the hinge and part of the non-canonical coiled-coil (1–370 N-terminal amino acids of Scy), interacted with T25-ParA, showing that the non-canonical coiled-coil domain was required for the ParA–Scy interaction and the N-terminal Scy domain did not inhibit this interaction. We believe that the full-length T18-Scy fusion protein does interact with T25-ParA, but the presence of long C-terminal domain and large size T18-Scy in comparison with T25-ParA affects T18-T25 proximity. Thus, our results collectively suggest that ParA interacts with Scy through its non-canonical coiled-coil domain.

To confirm the ParA–Scy interaction in *S. coelicolor* hyphae *in vivo*, we attempted to pull down the ParA–Scy complexes using affinity co-purification. These studies used strain M145 carrying pK48, which expressed *6his-scy* from a thiostrepton-inducible promoter in the multi-copy plasmid, pCJW93 [14]. The production 6His-Scy protein was induced and the protein purified from cell extracts using Ni-NTA affinity chromatography. SDS-PAGE analysis of the elution fractions revealed the co-elution of several proteins, one of which had an apparent size comparable with that of ParA (about 35 kDa; figure 1*b*). Western blotting using an anti-ParA antibody confirmed that ParA co-purified with 6His-Scy in this assay (figure 1*b*(ii)). A control experiment using strain BD11 containing an empty pCJW93 plasmid proved that the Scy–ParA interaction was specific (see the electronic supplementary material, figure S1). Thus, our results show that Scy and ParA interact in *S. coelicolor*.

### 3.2. The co-localization of Scy and ParA at the hyphal tip is transient

**Figure 2.**
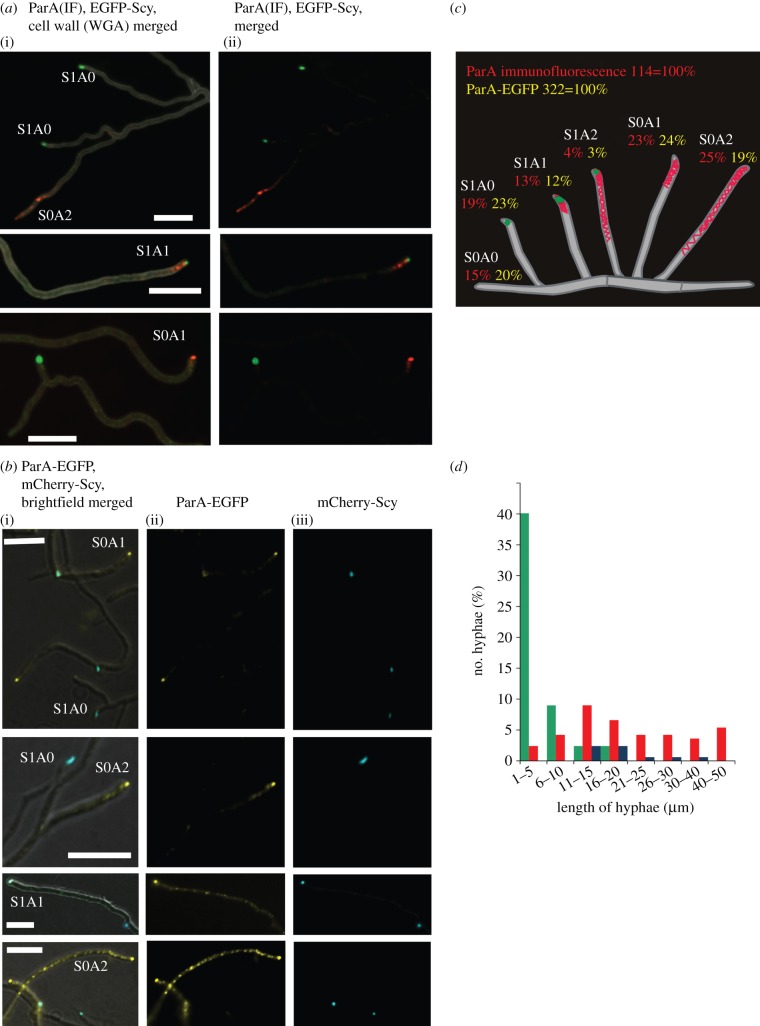
Co-localization of ParA and Scy. (*a*) Images showing aerial hyphae of a strain expressing *egfp-scy*; (i) merged fluorescence of cell wall staining with WGA-AlexaFluor 350 (grey), ParA immunofluorescence (red), EGFP-Scy (green), and (ii) merged fluorescence of ParA immunofluorescence (red) and EGFP-Scy (green). Alphanumeric abbreviations indicate hyphae with particular Scy and ParA signals, as described in the text and shown in (*c*). Scale bars, 5 μm. (*b*) Images showing aerial hyphae of a strain expressing *parA-egfp* and *mcherry-scy*; (i) fluorescence EGFP-ParA (false coloured yellow), mCherry-Scy (false coloured blue) merged with the Nomarski contrast image; (ii) EGFP-ParA (false coloured yellow); and (iii) mCherry-Scy (false coloured blue). Alphanumeric abbreviations indicate hyphae exhibiting particular fluorescent signal of Scy and ParA as described in the text and shown in (*c*). Scale bars, 5 μm. (*c*) Statistical analysis of Scy and ParA co-localization. Drawings of *Streptomyces* hyphae including percentages of aerial hyphae showing the particular patterns of ParA and/or Scy fluorescence, as indicated by the alphanumeric abbreviations. (*d*) Statistical analysis of the correlation between hyphal length and the ParA–Scy balance. Plot shows the frequency of occurrence of hyphae with specific lengths in relation to various ParA or Scy fluorescence signals. Green bar, Scy; red bar, ParA; blue bar, Scy + ParA.

Previous immunofluorescence experiments showed that ParA localized at the hyphal tip during vegetative growth [21]. In aerial hyphae, ParA was seen either as a comet-like assembly at the tip or, in longer hyphae, extending from the tip along the hyphae in the pattern that may be correlated with its suggested polymerization [21]. Scy was also localized at the tip; mCherry-Scy (expressed from translational fusion gene in addition to the wild-type *scy* gene, from the native *scy* promoter in pK57 integrative plasmid) co-localized with a C-terminal enhanced green fluorescent protein (EGFP) fusion of the polar landmark protein, DivIVA [14]. The tip localization and interaction of ParA and Scy prompted us to investigate the extent of ParA and Scy co-localization. We used two experimental approaches: ParA immunolocalization in strain M145 carrying pK56 (which produces EGFP-Scy), and co-localization of ParA-EGFP (produced instead of the wild-type protein from a fusion gene modified by chromosomal replacement) and mCherry-Scy (from the above-described pK57 integrative plasmid) in strain DJ595. The ParA-EGFP fusion protein is functional (3% of anucleate spores, in comparison with 26% in *parA* deletion mutant), and statistical analyses of the localization of ParA and Scy in both sets of experiments gave nearly identical results (figure 2*a,b*). In aerial hyphae, Scy was present in 36–38 per cent of tips, whereas ParA was detected in 58–66 per cent (localized as EGFP fusion or by immunofluorescence, respectively), reflecting the developmental induction of *parAB* transcription. This roughly 60 per cent frequency was somewhat less than the approximately 80 per cent previously reported for ParA in wild-type *S. coelicolor* [21], perhaps being the result of the presence of not fully functional fluorescent Scy fusion [14]. Alternatively, it could be a dosage effect, as EGFP-Scy or Scy-mCherry were produced alongside non-tagged Scy in the examined strains. Scy and ParA co-localized in only 15–17 per cent of the aerial hyphal tips analysed (figure 2*a*–*c*). In about three-quarters of the cases of co-localization, the ParA immunofluorescence was limited to the tip; in the remaining approximately 25 per cent, the Scy focus at the tip was accompanied by a ParA signal that extended along the hyphae. Thus, ParA and Scy co-localized in aerial hyphae, but the percentage of hyphae that showed co-localization was surprisingly low. On further analysis, we found a clear correlation between the length of the aerial hyphae and the presence of either ParA or Scy complexes (figure 2*d*). Scy was more readily detectable in the shorter (presumably actively growing) hyphae, whereas ParA was visible in the longer aerial hyphae, possibly coinciding with the developmental switch to sporulation. This developmentally dynamic pattern of Scy and ParA localization suggested that their presences in the tips could be mutually exclusive during certain differentiation stages of aerial hyphae.

### 3.3. Changes in the level of Scy affect ParA localization

**Figure 3.**
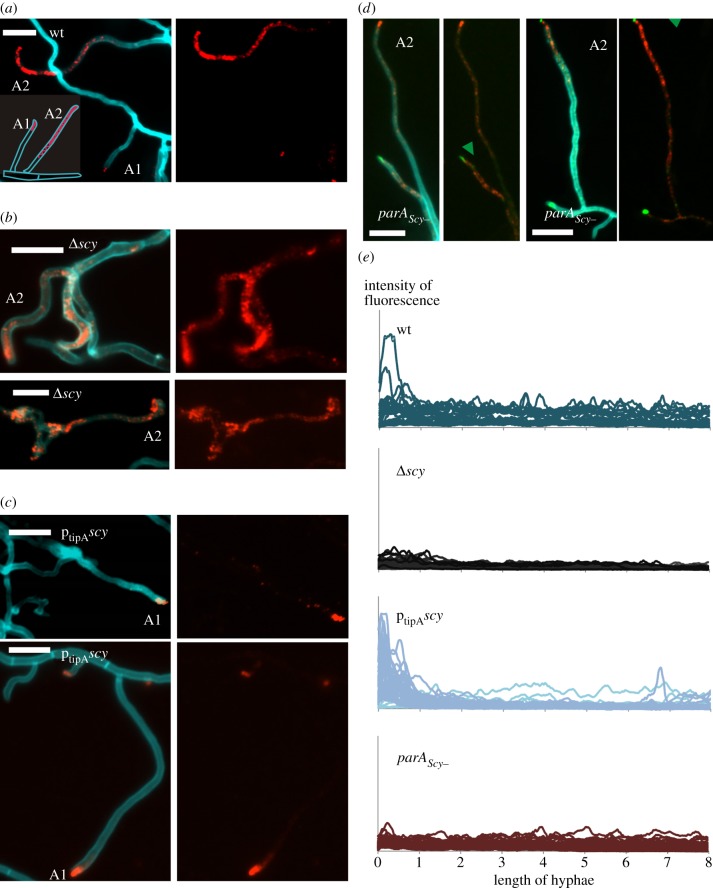
Influence of Scy level on ParA localization seen in images of aerial hyphae. Left panels: merged fluorescence of cell wall staining with WGA-AlexaFluor 350 (blue) and ParA immunofluorescence (red), and, in (*d*), Scy (green). Right panels: ParA immunofluorescence, in (*d*) overlain with Scy (green). Alphanumeric abbreviations indicate the ParA localization patterns given in the inset; green arrowheads mark Scy accompanying extended ParA_Scy−_; scale bars are 5 μm. (*a*) The M145 wild-type control showing ParA both tip-located and extended in hyphae. (*b*) The *Δscy* strain (K111) showing the ParA signal spread along hyphae. (*c*) The p_tipA_*his-scy* overexpression strain (M145pK48) showing ParA confined to hyphal tips. (*d*) The *parA_Scy−_* e*gfp-scy*-expressing strain (BD09) showing Scy co-occurring with the ParA signal. (*e*) Intensity profiles of ParA immunofluorescence along aerial hyphae in the indicated strains.

We hypothesized that if ParA and Scy undergo dynamic interactions, then alteration of Scy levels might affect ParA localization. Because the immunofluorescence of ParA was virtually identical to the localization pattern of ParA-EGFP, we used immunofluorescence to test our hypothesis in the *scy* deletion (K111) and overexpression (M145/pK48) strains. Holmes *et al*. [14] found that lack of Scy in strain K111 resulted in altered hyphal morphology and branching, with frequent occurrences of multiple abortive branches and uneven hyphal diameters in both vegetative and aerial hyphae. The branching aerial hyphae gave rise to spore compartments that were uneven in both size and DNA content, consistent with the dependence of ParA-driven chromosome segregation on Scy. Consistent with this, our immunolocalization experiments showed that the distribution of ParA was disturbed in the *scy* mutant hyphae. In contrast to the wild-type strain in which ParA fluorescence was confined only to the tips in some aerial hyphae (figure 3*a*,*e*), in the absence of Scy most of the hyphae, even very short ones, exhibited ParA immunofluorescence extending along the whole length of the apical compartments (48% of all hyphae analysed; figure 3*b*,*e*), whereas ParA signal exclusively at the tip was detected only in 4 per cent of hyphae (table 1). The spreading of the ParA signal from the hyphal tips in the absence of Scy may suggest that Scy plays an inhibitory role in ParA polymerization.

**Table 1.** Localization of ParA in *scy and parA mutant strains* (the experiments were performed using two different media and the differences were smaller than 5%)—percentage of hyphae with particular ParA localization pattern. *n* indicates the number of hyphae analysed.

ParA in aerial hyphae	at tips (%)	close to tips^a^ (%)	along hyphae (%)	no signal (%)
wild-type (M145) *n* = 172	15	33	29	19
*Δscy* (K111) *n* = 166	4	26	48	22
p_tipA_*his*_−_*scy* (M145pK48) *n* = 159	52	33	4	11
ParA_Scy−_ (BD08) *n* = 100	1	14	65	20

^a^See experimental procedures for detailed description.

If Scy inhibits spreading of ParA from the tip, a Scy-overproducing strain should show enhanced accumulation of ParA in the tip. We therefore studied ParA localization and the extension of ParA fluorescence along the hyphae in a Scy-overproducing strain (p_tipA_*scy* strain M145/pK48, in which *scy* was expressed form a thiostrepton-inducible promoter). Holmes *et al*. [14] previously monitored the effect of Scy overproduction by first growing the strain in the absence of the inducer for 10–14 h and then applying the inducer. As we wanted to study ParA localization during aerial growth, M145/pK48 was propagated in the continuous presence of the inducer. Under these conditions, the phenotypic defects were less pronounced than those observed after a pulse of induction. This may indicate that a burst of Scy overproduction can recruit DivIVA to ectopic locations, but fails to maintain this recruitment because DivIVA levels are unaltered during Scy overproduction, and thus quickly become limiting. In contrast to wild-type strain, where ParA was often found in the tips and extending along hyphae, the elevated levels of Scy in M145/pK48 strain were associated with distinct and strong ParA immunofluorescence signals confined to the hyphal tip region, with spreading along the hyphae seen in only 4 per cent of cases (figure 3*c*,*e* and table 1). ParA was found exclusively in relatively long hyphae, in which developmental induction of *parA* presumably occurred. These results demonstrate that changes in Scy levels affect ParA localization, possibly by affecting its polymerization.

### 3.4. Tip-proximal ParA localization depends on the interaction of ParA with Scy

**Figure 4.**
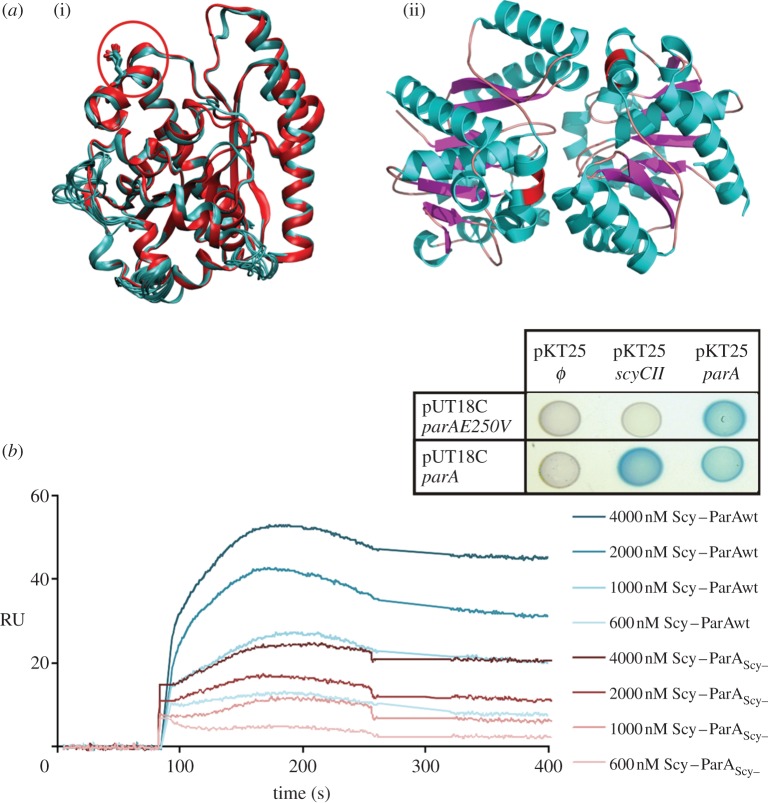
Identification of a ParA mutant (ParA_Scy−_) that is unable to interact with Scy. (*a*)(i) ten models of the *S. coelicolor* ParA structure superimposed over the structure of *T. thermophiles* ParA (PDB 1WCV), with E250 indicated by a red circle. (ii) Structure of the *T. thermophiles* ParA dimer (PDB 2BEK [5]), with the amino acids corresponding to *S. coelicolor* ParAE250 indicated by a red patch. (*b*) SPR analysis of the binding between Scy and ParA or ParA_Scy−_. ParA or ParA_Scy−_ (500 RU; response units) was immobilized on CM5 in the presence of ATP, and tested for an interaction with increasing concentrations of 6His-Scy. The background response signal from a control experiment was subtracted from the presented sensorgrams. Inset: Interactions of the ParAE250V mutant identified in the BTH system. Blue colonies indicate interactions of the analysed proteins.

In view of the findings that ParA and Scy interact directly, and that Scy is important for the correct localization and function of ParA, we hypothesized that the Scy–ParA interaction is involved in the cell-biological functions of ParA. To investigate this, we sought to identify and characterize a ParA mutant that was unable to interact with Scy. First, a library of random ParA mutants fused to T18 (see the electronic supplementary material) was screened for clones that retained the ability to dimerize, as shown by a positive BTH interaction when co-produced with T25-ParA. These clones were further screened for mutants that failed to interact with T25-ScyCII. Of 22 clones with the desired properties, nine contained a single mutation that generated the amino acid change E250V in the C-terminal domain of ParA. We modelled the structure of *S. coelicolor* ParA using the known structure of its homologue from *T. thermophilus* [5] (electronic supplementary material) and mapped the E250V mutation to an alpha helix located on the protein surface, close to the ParA dimerization interface (figure 4*a*).

To further characterize the effect of the mutation in the C-terminal alpha helix, we mutated the *parA* gene in the pGEX6P2-*parA* expression vector (and in the *S. coelicolor* chromosome; see below and in the electronic supplementary material). To facilitate the mutagenesis, we created a *Sna*BI restriction site in *parA*, which resulted in two amino acid changes (S249Y and E250V) in the putative Scy-interacting interface of ParA. Modelling indicated that the S249Y and E250V mutation should not destabilize the overall structure of ParA. The obtained mutant protein (ParAS249Y, E250V), designated ParA_Scy–_, was used in all following experiments. Similar to the ParAE250V protein identified in the BTH library screen, ParA_Scy−_ dimerized but did not interact with Scy in a BTH assay (figure 4*b*; electronic supplementary material, figure S2). Glutathione S-transferase (GST)-fused ParA_Scy−_was purified using affinity chromatography, and the GST tag was removed. The binding affinities of wild-type ParA and ParA_Scy−_ to 6His-Scy were compared using surface plasmon resonance (SPR). Purified recombinant wild-type and mutant ParA were immobilized on a CM5 chip in the presence of ATP, and an empty flow cell served as negative control for unspecific binding to the chip surface. Our analysis showed that Scy had a lower affinity to ParA_Scy−_ than to wild-type ParA (figure 4*b*).

Having shown that the mutation in the C-terminal alpha helix of ParA reduced its interaction with Scy, we constructed *S. coelicolor* strain BD08, in which the chromosomal *parA* was replaced with a mutated copy in the wild-type (M145) background. ParA_Scy−_ mutation had no obvious effect on growth and morphology, but microscopic analysis of DAPI- and wheat germ agglutinin (WGA)-stained hyphae revealed a chromosome segregation defect (21% anucleate spores and 36% mini-compartments) similar to that observed in the *parA* deletion mutant. This could arise from a weakening in the interaction with Scy or other interaction partners (e.g. ParB). The Western blotting analysis showed that the level of mutant protein is similar to the wild-type protein (see the electronic supplementary material, figure S1c, lanes 2 and 3), while the attempt to co-purify ParA_Scy−_ with 6His-Scy using Ni-NTA affinity chromatography from the strain BD10 (overproducing 6His-Scy from pCJW93p_tipA_*his-scy* plasmid in *parA*_Scy−_ background) confirmed that their interaction was weakened *in vivo* (see the electronic supplementary material, figure S1c, lanes E1 and E3). To determine whether ParA_Scy−_ localization still depended on Scy, we performed immunolocalization of ParA_Scy−_ in strain BD08. Interestingly, tip-localized ParA was visible in only 1 per cent of hyphae (compared with 15% in wild-type cells), whereas an extended ParA signal was observed in the majority of the analysed hyphae (65%; table 1). To confirm that a reduced interaction with Scy was at least partially responsible for the change in the behaviour of ParA_Scy−_, pK56 (expressing *egfp-scy*) was introduced into BD08 carrying the *parA* chromosomal mutation, generating strain BD09. In this strain, an extended ParA signal was often accompanied by a Scy focus at the tip (in 22% of analysed vegetative hyphae; figure 3*d*,*e*), whereas in M145pK56 expressing *egfp-scy* in the wild-type background, the fraction of such hyphae was much lower (4%).

Taken together, these results strongly indicate that the interaction of ParA with the Scy protein plays an important part in the transition of ParA between its tip-associated and extended localization. Thus, the enhanced and premature extension of ParA_Scy−_ observed in aerial hyphae that contained Scy foci at their tips could result from a weakening in the interaction with Scy. Our analysis of the ParA_Scy−_ mutant therefore suggests that an efficient interaction with Scy is required to inhibit the premature polymerization of ParA in aerial hyphae.

### 3.5. Scy and ParA each affect polymerization of the other

As our microscopy studies suggested that Scy might negatively influence the polymerization of ParA along the hyphae, we tested this hypothesis *in vitro* using two methods: a pelleting assay and measurements of dynamic light scattering (DLS).

**Figure 5.**
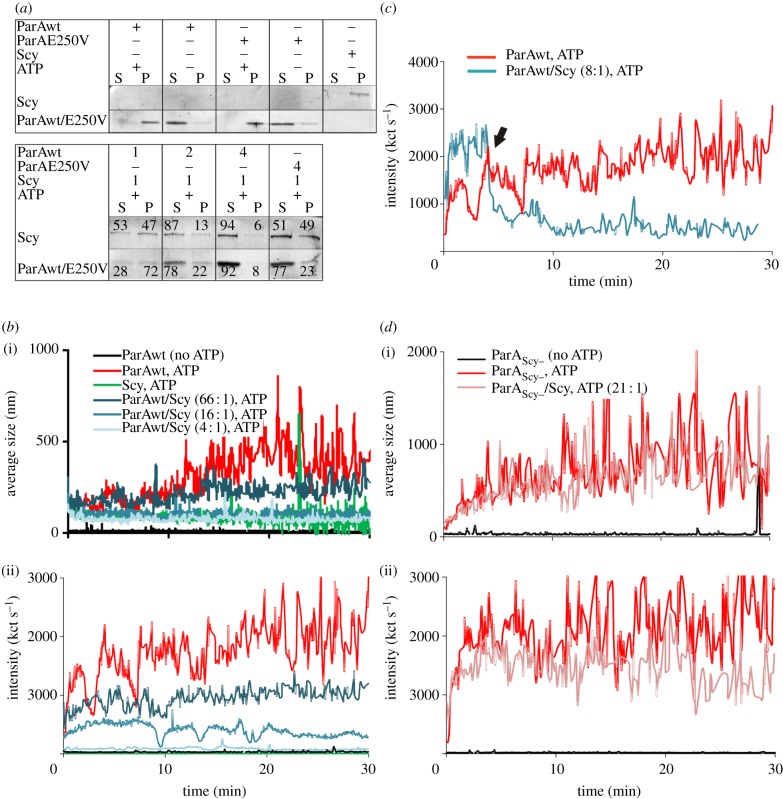
Influence of Scy on ParA and ParA_Scy−_ polymerization. (*a*) Pelleting assay. (i) Polymerization of ParA (4 μM) or ParA_Scy−_ (4 μM) in the presence of ATP and 6His-Scy (1 μM), as indicated. P, pellet fraction/polymerized protein; S, soluble fraction. (ii) The effect of increasing ParA concentration (1–4 μM) on Scy polymerization (1 μM), with the ratio of ParA to Scy indicated. The presented image is a representative example of triplicate experiments, and the numbers in (ii) are the average quantifications (with s.d. less than 10%) of the signals in each lane. (*b*) Measurements of dynamic light scattering (DLS). ParA polymerization is inhibited in the presence of 6His-Scy. (i) Average polymer size (nm). (ii) Changes in light-scattering intensity, expressed as kct s^−1^. (*c*) Measurements of DLS. ParA depolymerization is induced by addition of 6His-Scy. Plot shows the changes in light-scattering intensity, expressed as kct s^−1^. The arrowhead marks the time at which 6His-Scy was added to the polymerized ParA. (*d*) Measurements of DLS. There is no difference in polymerization of ParA_Scy−_ in the presence and absence of 6His-Scy. (i) The average polymer size (nm). (ii) Changes in light-scattering intensity, expressed as kct s^−1^.

The pelleting assay revealed that ParA in the presence of ATP was predominantly found in the pellet fraction (figure 5*a*), suggesting that it forms higher-order complexes. Scy also was found in the pellet fraction, but unlike ParA, the polymerization of Scy did not require ATP. When Scy (1 μM) and ParA (1 μM) were mixed in the presence of ATP, both proteins appeared in both the soluble and pellet fractions, indicating that there had been reciprocal inhibition of higher-order assembly. What is more, the higher the ratio of ParA to Scy, the greater the proportion of Scy in the supernatant was observed (figure 5*a*).

As we found that the interaction of ParA_Scy−_ with Scy was reduced in comparison with that of wild-type ParA, we speculated that the mutant ParA should be less effective in interfering with Scy polymer assembly, and that its own polymerization should be less susceptible to Scy-mediated inhibition. ParA_Scy−_ (4 μM) polymerized similarly to wild-type ParA in the presence of ATP (figure 5*a*; electronic supplementary material, figure S3), indicating that its intrinsic oligomerization properties were not significantly perturbed by the mutation. However, its polymerization was less affected by the presence of Scy, and the influence of ParA_Scy−_ on Scy polymerization was diminished compared with wild-type ParA, as shown by the decreased proportion of Scy in the soluble fraction (figure 5*a*). These results suggest that an efficient interaction between Scy and ParA is required for their reciprocal inhibition of higher-order assembly or polymerization.

We next assessed ParA filament assembly/disassembly in the presence of Scy in real time using DLS, which assesses the abundance and size of particles. We could not use this method to study Scy polymerization because Scy aggregates were not detected in DLS (figure 5*b*), presumably owing to the physical properties of the assemblies. Consistent with a previous report [24], the average intensity of light scattering by ParA (2.5 μM) aggregates formed in the presence of ATP was approximately 2000 kilocounts per second (kct s^−1^), whereas the particle size was 500 nm. When incubated with Scy and ATP, ParA failed to polymerize efficiently; both light-scattering intensity and particle size were dose-dependently reduced by Scy, to about 84 kct s^−1^ and 80 nm, respectively, in the presence 0.6 μM Scy (ParA : Scy ratio 4 : 1). Thus, Scy inhibited ATP-dependent ParA polymer formation (figure 5*b*). Moreover, the addition of 6His-Scy (0.3 μM) to prepolymerized ParA filaments led to an immediate decrease of light scattering and particle size, indicating rapid polymer disassembly (figure 5*c*). This inhibition of polymerization was not observed when ParA_Scy−_ (3.3 μM) was analysed; in these experiments, the light-scattering readings were independent of addition of Scy (figure 5*d*).

In summary, our *in vitro* analyses collectively suggest that Scy inhibits ParA polymerization and triggers depolymerization of ParA.

### 3.6. The interplay of ParA and Scy coordinates growth cessation and chromosome partitioning

**Figure 6.**
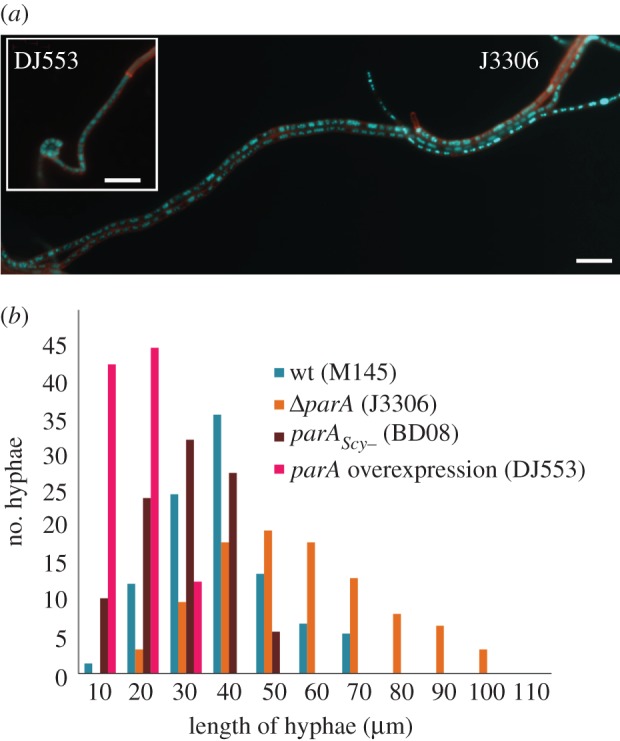
Length comparisons of prespore chains from wild-type, J3306 (*parA* deletion), DJ553 (*parA* overexpression) and BD08 (*parA_Scy−_*). (*a*) Example images of the aerial hyphae of *ΔparA* strain (J3306) and *parA* overexpression DJ553 (inset), stained with DAPI and WGA-Texas Red. Scale bar, 5 μm. (b) The average hyphal lengths were 44, 62, 21 and 33 µm, respectively, as measured in 73 (wt), 61* (*ΔparA*), 87 (*parA* overexpression) and 87 (ParA_Scy−_) hyphae. Asterisk indicates that the calculated average length may be lower than actual because the long chains of prespores were difficult to measure.

The influence of Scy on ParA polymerization observed *in vitro* probably underlies the irregular septation and chromosome segregation in the *scy* mutant. As Scy polymerization has been implicated in hyphal growth, the reciprocal effect of ParA on Scy assembly suggested by polymerization assays could be predicted to disturb elongation of the aerial hyphae in the *parA* mutant. To investigate this possibility, we analysed the lengths of prespore chains (septated aerial hyphae that have stopped growing but have not yet turned into easily dispersing spores) in the wild-type, *parA* deletion (J3306), *parA* overexpression (DJ553) and *parA_Scy−_* (BD08) strains. We speculated that *parA* deletion should result in more persistent Scy assemblies and the formation of longer aerial hyphae, whereas ParA overexpression should induce earlier disassembly of Scy complexes and earlier inhibition of hyphal extension. These expectations were met (figure 6). The average length of septated hyphae in wild-type cells was 44 µm, and most of them (75%) were between 30 and 60 µm. In the *parA* deletion strain, many very long hyphae that were not confined in the view field were observed (figure 6*a*). The average length of those which were visible in the whole length was 62 µm, and 50 per cent of the observed hyphae were longer than 60 µm (compared with 11% in the wild-type). In the *parA* overexpression strain, the average length of hyphae was 21 µm, and all observed hyphae were shorter than 40 µm. In the ParA*_Scy_*_−_-producing strain, we speculated that the premature formation of polymers (owing to their partial immunity to Scy-mediated depolymerization) should result in earlier partitioning and sporulation septation, whereas less efficient ParA-mediated disassembly of the Scy complex could promote extensive growth of hyphae. Contrary to the latter expectation, the aerial hyphae of BD08 strain were short, with an average length of 33 µm, and 25 per cent of the observed hyphae were shorter than 30 µm (compared with 14% in the wild-type strain; figure 6). This may suggest that formation of ParA polymers triggers some control mechanisms that inhibit further hyphal growth.

In summary, the obtained results show that the interplay of ParA and Scy may be involved in coordination of the cessation of aerial growth and the onset of DNA partitioning.

## 4. Discussion

The bacterial ParAs are regarded as cytoskeletal proteins owing to their functions and dynamic polymerization properties. Recent studies provided the first insights into their involvement in the active segregation of bacterial chromosomes, but we still know relatively little about the factors responsible for regulation of their dynamics. The *Streptomyces* ParA protein shares most of the biochemical properties of the other ParA homologues, but it exhibits a very complex localization pattern related to the developmental stage of the hyphae [21]. We hypothesized that *Streptomyces* ParA requires growth-stage-specific regulators, and thus searched for its interaction partners. Here, we provide evidence that the initiation of ParA filamentation is regulated by Scy, a polarisome protein that is involved in the regulation of tip elongation and branching.

### 4.1. ParA interacts with the non-canonical coiled-coil C-terminal domain of Scy

Here, we show for the first time that the *Streptomyces* chromosome partitioning protein, ParA, interacts with the tip-associated polarity protein, Scy. BTH subdomain analysis of the ParA–Scy interaction showed that ParA binds through the C-terminal domain of Scy, which was previously reported to exhibit a regular non-canonical coiled-coil structure based on a novel repeat of 51 aa residues forming 14 supercoiled turns (CC51 [12]). A predicted hinge region of eight disordered aa residues separates the C-terminal domain from a shorter canonical coiled-coil domain (CC7) in the N-terminal fragment of the protein. Interestingly, Scy was previously shown to dimerize, and the N-terminal and C-terminal domains were proposed to provide two independent dimerization interfaces, leading to the hypothesis that Scy assembles into a long ‘rope’ formed by the two coiled-coil domains [12]. This formation of higher-order structures by Scy has been confirmed by pelleting assays [14]. Thus, our present results and the previous findings suggest a model in which the interaction between ParA and the C-terminal domain of Scy is likely to inhibit the higher-order organization of Scy.

### 4.2. Scy is a novel regulator of ParA polymerization

Our *in vitro* polymerization studies established that Scy is a novel negative regulator of ParA filamentation, and suggested that this effect may be reciprocal. Scy blocks ParA polymerization and induces its depolymerization, whereas ParA inhibits the assembly of Scy into higher-order structures. Two other proteins are known to interact with ParA in *S. coelicolor*: the first is the Actinomycete-specific protein, ParJ, which triggers the disassembly of ParA polymers *prior to* sporulation septation, apparently via the ParJ-mediated stabilization of ParA monomers [24]; and the second is ParB, which enhances the ATPase activity of ParA [21]. ParB homologues have been shown to affect the polymerization of cognate ParAs by modulating their ATPase activities [5,26–29]. In contrast to the other sufficiently characterized ParA homologues, the *Streptomyces* ParA protein requires ATP, but not DNA for its *in vitro* polymerization [21]. The unique biochemistry of *Streptomyces* ParA polymer assembly may be related to the requirement for long ParA filaments that extend over tens of micrometres in the aerial hyphae and govern the distribution of tens of ParB nucleoprotein complexes. Moreover, the complex spatio-temporal localization pattern of *Streptomyces* ParA, which is highly dependent on the developmental stage, is likely to involve a complex regulatory system that governs ParA polymerization. Spatial regulation of ParA filamentation was recently shown in *C. crescentus*; in this system, a novel regulator of ParA polymerization, the coiled-coil pole protein, TipN, is required to maintain the directionality of the transfer of the ParB–DNA complex towards the new cell pole, probably via ParB-mediated titration of depolymerized ParA. TipN was hypothesized to nucleate or stabilize ParA structures at the new pole, or, alternatively, by binding ParA, to increase its local concentration and bias the insertion of free ParA molecules into the structure at the new pole [7,8].

### 4.3. Glutamate 250 of ParA is crucial for its interactions with Scy

By screening a ParA mutant library in the BTH system, we identified the interaction interface in ParA and showed that glutamate residue 250 is required for its interaction with Scy. Although the ParA_Scy−_ mutant retained its abilities to dimerize and oligomerize, Scy had a diminished effect on the polymerization of the mutated protein, and the immunolocalization of ParA_scy−_ along the hyphae differed from that of wild-type ParA. Based on the structure of the *T. thermophilus* homologue of ParA [5], glutamate 250 is part of a C-terminal helix located at the protein surface close to the dimerization interface, and it may be involved in bond formation with one of several arginines in the C-terminal part of ParA. The E250V mutation dramatically influenced the electrostatic potential on the surface of the ParA protein (see the electronic supplementary material, figure S4). As ParA forms a sandwich dimer [5], nucleotide exchange may require disruption of the dimer and may not occur in the filamentous form. Thus, Scy-mediated regulation of polymerization could involve its interaction with the polymer or dimer forms of ParA, or else Scy might bind ParA monomers and inhibit their dimerization. Our failure to observe any interaction between Scy and the dimerization-deficient ParAK44E mutant [21] (electronic supplementary material, figure S2) indicates that Scy is likely to interact with polymers or dimers.

### 4.4. Scy can recruit ParA to the growing tip

As part of the TIPOC, Scy is important for the assembly, localization and organization of this polarity centre, and can recruit other TIPOC proteins, such as DivIVA, to emerging tips [14]. In the present study, the direct interaction observed between Scy and ParA *in vitro*, and the co-localization of ParA and Scy at some hyphal tips, indicate that ParA is a Scy-recruited protein. Other observations reinforce this idea: Scy overproduction resulted in large ParA foci at hyphal tips; most hyphae in the *scy* deletion mutant exhibited ParA filamentation along the hyphae, with few branches (4%) containing ParA restricted to the tip; and Holmes *et al*. [14] found very uneven chromosome distributions in the spores of the *scy* mutant. Moreover, the excessive ParA polymerization of the *scy* mutant was not sufficient for correct chromosome segregation, suggesting that either the initial anchoring of ParA at the tip or the precise timing of ParA polymerization is important and Scy-dependent. ParA and Scy overlapped in only a minority of the analysed hyphal tips, suggesting that ParA recruitment may be transient, perhaps only occurring at the beginning of *parA* induction in actively growing hyphae, *prior to* chromosome segregation. Notably, most ParA homologues localize close to the cell poles [7,9,30]. In *C. crescentus*, polar extension of ParA is dependent on tip-associated TipN. Although chromosome segregation in sporulating *Streptomyces* hyphae does not occur uni- or bidirectionally, as in rod-shaped bacteria, and nor does it involve the pulling of single ParB complexes, the formation of ParA polymers is crucial for the efficient segregation of numerous chromosomes in apical aerial hyphal compartments.

### 4.5. The correlation of the balance between Scy and ParA with the growth of hyphae

**Figure 7.**
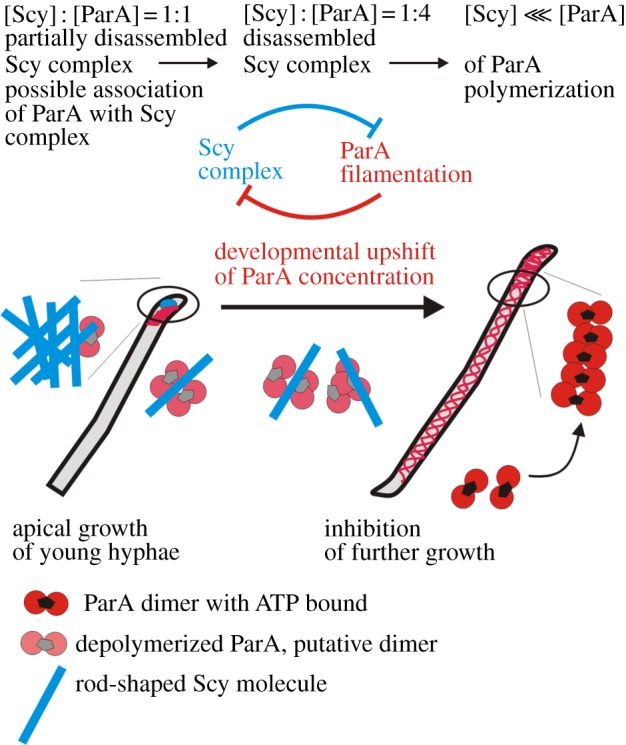
Model of the ParA–Scy interaction in *Streptomyces* hyphae.

Our results illuminate the connection between bacterial chromosome dynamics and the molecular mechanisms that impose polarity. Scy is part of the TIPOC for polarized tip extension [14], and ParA was previously reported to form structures extended from the tips of aerial hyphae *prior to* the formation of ParB complexes, so we expected to observe partial co-localization of these proteins at the hyphal tips. Such co-localization was observed, but only infrequently, suggesting that it may be transient. The lack of evidence for stable complex formation *in vivo* seems to corroborate the negative effects of Scy on ParA polymer assembly observed in our pelleting assays, which may possibly be reciprocal. Moreover, this dynamic interplay was correlated with hyphal length: shorter (presumably actively growing) hyphae contained strong Scy foci; ParA foci were present in the long filaments that were most likely to be switching from growth to sporulation; and the two proteins were both present (at varying amounts and ratios) in the tips of intermediate-length hyphae. Coupled with results showing that changes in Scy levels had clear effects on ParA localization, these data suggest that Scy inhibits premature polymerization of ParA, thereby preventing premature chromosome segregation (figure 7). Developmental induction of the *parAB* operon [31] probably increases ParA to a level that escapes Scy-mediated negative regulation as hyphae reach their optimal length for sporulation. Upon release from Scy inhibition, ParA begins polymerizing along the hyphae, which is followed by chromosome condensation. This escape was overridden by overexpression of Scy, whereas enhanced ParA polymerization was observed in the absence of Scy and when ParA was mutated to a form whose interaction with Scy was significantly inhibited.

The ParA : Scy ratio shifts towards ParA at the time of transition from rapid growth of aerial hyphae to further stages of sporulation; this arises from the developmentally regulated and aerial-hyphae-specific induction of the *parAB* promoter [31]. We hypothesize that Scy depolymerization bringing about the cessation of further hyphae elongation may be triggered by increased concentration of ParA (figure 7). This could explain shorter hyphae of ParA overexpression strain and longer of *parA* deletion strain. The developmental upshift in *parAB* expression is very strongly dependent on two developmental genes, *whiA* and *whiB*, and the WhiA protein has been shown to interact with the *parAB* promoter region [31,32]. Consistent with our suggestion that high ParA levels may lead to disassembly of Scy complexes and growth cessation, mutations in *whiA* and *whiB* result in unusually long, non-sporulating aerial hyphae [33]. Our model is additionally supported strongly by the observation made in *Streptomyces venezuelae—*the model organism that is able to sporulate in liquid culture and whose differentiation can be followed in the time lapse experiment [34]. The microscopy analysis of *S. venezuelae* ParA-EGFP localization showed close correlation of appearance of ParA fluorescence with hyphal growth cessation (see the electronic supplementary material, figure S5).

In summary, we have identified a novel regulator of bacteria-specific cytoskeletal protein ParA. The presented model describes the control of ParA polymerization by the Scy complex in the growing hyphae. It also explains how the shift of the balance between ParA and Scy could lead to destabilization of Scy assembly and thereby could coordinate the growth cessation. Our results illuminate for the first time the direct link between chromosome dynamics and mechanisms that govern polar cell growth in bacteria.

## 5. Material and methods

### 5.1. Bacterial strains and growth conditions

The *S. coelicolor* and *Escherichia coli* strains are listed in the electronic supplementary material, table S1. Culture conditions, antibiotic concentrations, transformation protocols and conjugation methods followed generally accepted procedures for *E. coli* [35] and *Streptomyces* [36].

### 5.2. Protein purification

Recombinant 6His-Scy was overexpressed in *E. coli* BL21(DE3) carrying pGS2 (see the electronic supplementary material, table S1) induced with 0.1 mM isopropyl β-D-1-thiogalactopyranoside (IPTG) for 3.5 h at 30°C. The protein was purified from the soluble fraction by metal-ion affinity chromatography on a HisTrap HP column (Sigma) according to the manufacturer's recommended procedure, followed by ion exchange chromatography using HiTrap (monoQ [14]). GST-fused ParA_Scy−_ was produced in *E. coli* BL21(DE3) induced with 0.1 mM IPTG overnight at 16°C, and purified according to the previously described protocol for ParA [21].

### 5.3. ParA–Scy co-purification

For co-purification of Scy and ParA, *S. coelicolor* strains were grown in liquid tryptic soy broth–polyethylene glycol (TSB-PEG) medium (21 h, 30°C) and 6His-Scy production was induced with thiostrepton (final concentration 20 μg ml^−1^) for 5 h. The pellets were washed twice with buffer A (50 mM NaH_2_PO_4_, pH 8.0, 500 mM NaCl and 10 mM imidazole). Cell extracts were generated by sonication, cell debris was removed by centrifugation and the supernatants were subjected to nickel affinity chromatography (HisTrap HP, 1 ml; GE Healthcare) using fast performance liquid chromatography (FPLC). Non-specific proteins were removed by washing samples with 50 mM NaH_2_PO_4_, pH 8.0 and 500 mM NaCl containing 15 mM imidazole, and 6His-Scy was eluted using an imidazole gradient (15 nM–300 mM). Fractions (1 ml each) were collected and analysed using SDS-PAGE and Western blotting with an anti-ParA antibody.

### 5.4. Bacterial two-hybrid system and library screening

Application of the BTH system [25] and library screening were performed as previously described [24]. Details of the construction of the *parA* mutant library in the BTH system, and the screening for ParA mutants that failed to interact with Scy are provided in the electronic supplementary material.

### 5.5. Construction of mutant strains

PCR targeting [37] was used to construct the mutant strains (see the electronic supplementary material, table S1). Details are given in the electronic supplementary material.

### 5.6. Microscopy

For microscopy, sample strains were grown for 42–44 h on coverslips inserted in minimal medium (MM) agar containing 1 per cent mannitol, or on cellophane membranes laid on SFM. For Scy induction, the strain was grown in the presence of thiostrepton (10 µg ml^−1^) on MM or soya flour mannitol (SFM), or grown for 40 h on SFM, and then transferred to an SFM plate containing 20 µg ml^−1^ of thiostrepton. Immunofluorescence staining and cell wall staining with WGA was performed as described previously [21]. Fluorescence microscopy was carried out using a Zeiss AxioImager M1 with an AxioCamMRm Rev. v. 2 camera. Images were analysed using the AxioVision software equipped with AutoMeasure module. For analysis of ParA immunofluorescence, the signal intensities along the hyphae were measured using AxioVision. Hyphae were classified as exhibiting tip-proximal ParA localization if the signal intensity dropped more than twofold along the hyphal length.

### 5.7. Polymerization studies

SPR analysis, pelleting assays and monitoring of polymerization were performed as previously described [24]. For details, see the electronic supplementary material.

## 6. Acknowledgements

This work was supported by the Ministry of Science and Higher Education (grant no. N N301 285437). B.D. was supported by a scholarship from the President of the Polish Academy of Sciences and the British Council Young Investigator Programme. We thank Andrew Hemmings for helping with the DLS measurements, Agnieszka Ptasińska for helping with the pelleting assays and Justin Nodwell for providing the *S. coelicolor* expression library in the BTH system. We are also very grateful to Keith Chater for providing critical comments on the manuscript.

## Supplementary Material

Supporting Information

## Supplementary Material

Supporting Figures
