# Supplementary material for: Dynamic interplay of ParA with the polarity protein, Scy, coordinates the growth with chromosome segregation in Streptomyces coelicolor
Source: Open Biol. 2013 Mar;3(3):130006. doi: 10.1098/rsob.130006 (PMC3718342; doi:10.1098/rsob.130006)
Supplement: Supporting Information [file rsob130006-s1.pdf]

Supplementary Materials and Methods

Bacterial two hybrid (BTH) system and library screening

Briefly a construct expressing *parA* in fusion with the C terminus of the T18 fragment of adenylate cyclase in *E. coli* BTH101 [1] was used to screen an *S. coelicolor* genomic library in pT25 kindly provided by Dr Justin Nodwell, McMaster University, Hamilton, Canada. The constructs containing fragments of *scy* gene were obtained by cloning PCR products obtained with the primers listed in Table S2. The fragments were cloned using restriction sites as indicated, and sequenced and used together, when appropriate, with the plasmids, pUT18c-Scy, pUT18c-ScyC and pUT18c-ScyN (Table S1) [2]. The *parA* mutant library in the BTH system was constructed using DNA fragments containing randomly mutated *parA*, obtained using Diversify PCR Random Mutagenesis (Clontech) and primers ETH_SA_F and ETH_SA_R. The fragments after digestion with XbaI and KpnI were ligated into pUT18C. The ligation mixture was used to transform *E. coli* DH5α to obtain 3000 independent colonies. Plasmid DNA isolated from the pooled colonies was used as the library in screening for clones producing mutant T18ParA* which did not interact with T25ScyCII. From about 4600 BTH101 colonies co-expressing T18ParA* and T25ScyCII about 1000 were white on the McConkey plates, indicating lack of interaction. They were re-streaked on the new plates and subjected to total plasmid DNA isolation. The obtained plasmid mixture after digestion with BglII to linearise pKT25ScyCII, was used to transform BTH101 containing pKT25*parA*. This step enabled identification of the clones expressing mutated *parA* which retained the ability to dimerize. Among about 7400 colonies 500 were red on the McConkey plates and after re-streaking them to new plate the total plasmid DNA was extracted from them. To create a sub-library for the second round of selection the plasmid preparation was digested with XbaI and KpnI, ligated to pKT25 and used for transformation of DH5α. The resulting pKT25parA* sub-library was used to transform BTH101pUT18C*scyCII.* After selection of white colonies (about 300) the next round of enrichment was performed as described above, and finally the 22 library clones that gave a negative signal with ScyCII but positive with ParA were sequenced. Eight of the sequenced clones contained a single mutation in *parA* gene resulting in amino acid exchange E250V.

Construction of mutant strains

The PCR-targeting procedure [3] was employed to replace *parA* gene with *parA-egfp* fusion gene and *parA* gene with a *parA* containing mutations S249Y,E250V. The strain expressing *parA-egfp* was constructed by knock in of a cassette encoding *egfp* and apramycin resistance gene flanked by NdeI sites. Since *parA* stop codon and *parB* start codon overlap, the care was taken not to influence the translation of *parB*. The cassette was amplified using primers ParAgfp_fw and A_gfp09R (Table S2) and the resulting PCR product was used to target the cosmid H24 in arabinose-induced *E. coli* BW25113/pIJ790, inserting the cassette downstream of *parA* gene. Cosmid H24*parA egfp apra* was

digested with NdeI to remove apramycin resistance cassette and to ligate oligonucleotide (Table 2) with the sequence of 3' end of *parA* gene containing potential RBS site for *parB*. Subsequently the *bla* gene in the SuperCos part of cosmid H24*parA-egfp* was exchanged for an *apra-oriT* cassette, yielding H24*parA-egfp*apra-oriT. This construct was used for conjugation into *S. coelicolor* J2538 [*parAB::apra*] [4] . Kan^R exconjugants were screened for the loss of both Kan^R and $Apra^R$, indicating a double-cross-over allelic exchange of the *parAB* locus of J2538 giving strain DJ590. Mutation of the *parA* gene and the lack of influence on expression of *parB* was verified by sequencing and Western blotting.

In order to construct the mutant $parA_{scy-}$ strain, the apramycin resistance cassette deriving from plasmid pIJ773 was amplified using primers YVmutfw and YVmutrv (Table S2). The resulting PCR product flanked with SnaBI sites was used to target the cosmid H24 in arabinose-induced *E. coli* BW25113/pIJ790, inserting the cassette in the part of *parA* coding for S249-E250. Cosmid H24*parA::apra(E250)* was digested with SnaBI and religated to remove the apramycin resistance cassette and to restore a full-length *parA* gene with a SnaBI site encoding a mutated protein with two amino acids exchanged *S249Y,E250V*. Subsequently the *bla* gene in the SuperCos part of cosmid H24*parA(E250V)* was exchanged for an *apra-oriT* cassette, yielding H24*parA(E250V)*apra-oriT. This construct was used for conjugation into *S. coelicolor* J2538 [*parAB::apra*] [4]. Kan^R exconjugants were screened for the loss of both Kan^R and $Apra^R$, indicating a double-cross-over allelic exchange of the *parAB* locus of J2538 giving strain BD08. Mutation of the *parA* gene was verified by sequencing.

Surface plasmon resonance (SPR) analysis

For SPR analysis, the same amount of untagged ParA or ParAE250V protein (400 response units [RU]) was immobilized on the CM5 Sensor Chip in the presence of ATP, according to the manufacturer's instructions. As a control, a flow channel without immobilised protein was used. SPR analysis was performed on a BiaCore 3000, by applying solutions of 6His-Scy in increasing concentrations (as indicated) in binding buffer (10 mM HEPES/KOH, pH 7.4, 150 mM NaCl, 50 μg/ml BSA, 5 mM $MgCl_2$, 0,05% Tween 20). Binding was measured for 3 min at flow rate 20 μl/min at room temperature in SPR running buffer (10 mM HEPES/KOH pH 7.4, 150 mM NaCl, 0.05% Tween 20). The Scy injections were followed by two to four injections of 1-5 M NaCl to remove all bound analyte from the sensor chip. The results were plotted as sensograms after subtraction of the background response signal obtained in a control experiment (empty channel).

Pelleting Assay

For pelleting assays, from 1 to 4 μM ParA or 4 μM ParAE250V and 1 μM 6His-Scy were incubated in reaction buffer in a total of 40μl (50mM Tris-HCl, pH 7.0, 150 mM NaCl, 5 mM $MgCl_2$) in the presence of ATP (2 mM). After 5 min preincubation at RT and incubation at 30°C for 30 min the samples were centrifuged at 20°C at 100 000 rpm for 30 min in a TLA100 rotor (Beckman, ultra TLX). The supernatants were removed and mixed with gel-loading buffer. The pellets were

resuspended in 15-20 μl of reaction buffer and mixed with gel-loading buffer. The whole pellet fraction and half of the supernatant fraction were subjected to SDS-PAGE and stained with Coomassie blue.

Dynamic light scattering (DLS)

Polymerisation of ParA (purified as described earlier, cleaved from GST-fusion [5] in the presence of Scy was measured by DLS (DynaPro Wyatt Technology). To observe the effect of Scy on ParA and ParAE250V polymerisation, 14 μl of protein mixture (2,5 μM ParA mixed with 0,6, 0.15 or 0.04 μM 6His-Scy and 3.33 mM ParAE250V mixed with 0.15 6His-Scy in 50 mM Tris-HCl pH 7.0, 150 mM NaCl, 5 mM $MgCl_2$) was preincubated at room temperature for 5min. Then ATP (2mM) was added to the protein solution, which was placed in the quartz cuvette and incubated at 30°C in the DynaPro chamber. In a complementary approach 6His-Scy (0.3 μM) was added after 5 min of ParA incubation in the presence of ATP at 30°C in the chamber. Measurements were taken every 2 s.

Modelling of *Streptomyces coelicolor* ParA structure

The ParA sequence was aligned to the five best available PDB templates (1WCV, 2BEJ and four independent subunits of 2BEK), all representing the same Soj chromosome segregation protein from *Thermus thermophilus*. The sequences were aligned using ClustalW [6] using optimal pairwise alignment algorithm and the default scoring scheme (Gonnet scoring matrices, 10/0.1 pairwise affine gap penalties and 10/0.2 for MSA) but with exhaustive iterative realignment at every step. As the basis for structure modelling, two sequence alignments were prepared: MSA1 included only the highly similar *T. thermophilus* Soj structures, and MSA2 included also sequences of plasmid partition protein 3EZ2, 3EZ7 and 3EZ9.

The Modeller program by Andrey Sali [7] was used for the homology-based modeling the ParA structures. Models based on MSA1 were of higher quality and they were chosen for further work. Using settings advised for a thorough model refinement (simulated annealing refinement with up to 300 steps of VTFM optimization), prepared were 10 initial models. They all are missing the 28 N-terminal residues and 22 C-terminal ones, due to lack of any structural template in these parts. All 10 structures produced follow the fold of the templates very closely, apart from some fragments with short insertions, where a variety of conformations appears.

The best among these 10 models was equilibrated over 1ns of molecular dynamics in explicit water with Na^+ and Cl^- ions neutralizing the protein charge and providing the salt concentration of 0.15M. Molecular dynamics simulation was performed in NPT ensemble, standard conditions, using CHARMM forcefield [8] . The equilibrated WT structure was used to introduce E250V mutation and the mutant was also subject to MD equilibration for 1ns. Both structures exhibit stable RMSD deviation over time, between 0.7-0.8Å for WT and 0.8-1Å for E250V, indicating structural stability of the models with slight structural differences between WT and the mutant. Electrostatic potential was calculated from CHARMM atomic charges [9] using APBS on the basis of Poisson-Boltzmann model

[9] and visualized on the protein surface with VMD [10]. The comparison between surface electrostatic potential of both wild type and E250V models is presented in Fig. 2S. Calculations have been carried out in Wroclaw Centre for Networking and Supercomputing (http://www.wcss.wroc.pl).

References

1. Karimova G, Pidoux J, Ullmann A, Ladant D (1998) A bacterial two-hybrid system based on a reconstituted signal transduction pathway. *Proc Natl Acad Sci U S A* 95(10):5752-5756.
2. Walshaw J, Gillespie MD, Kelemen GH (2010) A novel coiled-coil repeat variant in a class of bacterial cytoskeletal proteins. *J Struct Biol* 170(2):202-215.
3. Gust B, Challis GL, Fowler K, Kieser T, Chater KF (2003) PCR-targeted Streptomyces gene replacement identifies a protein domain needed for biosynthesis of the sesquiterpene soil odor geosmin. *Proc Natl Acad Sci U S A* 100(4):1541-1546.
4. Kim HJ, Calcutt MJ, Schmidt FJ, Chater KF (2000). Partitioning of the linear chromosome during sporulation of *Streptomyces coelicolor* A3(2) involves an *oriC*-linked parAB locus. *J Bacteriol.* 182, 1313-1320.
5. Jakimowicz D, Zydek P, Kois A, Zakrzewska-Czerwinska J, Chater KF (2007) Alignment of multiple chromosomes along helical ParA scaffolding in sporulating Streptomyces hyphae. *Mol Microbiol* 65(3):625-641.
6. Larkin MA, Blackshields G, Brown NP, Chenna R, McGettigan PA, McWilliam H, Valentin F, Wallace IM, Wilm A, Lopez R, Thompson JD, Gibson TJ and Higgins DG (2007) Clustal W and Clustal X version 20. *Bioinformatics* 23(21): 2947-2948
7. Sali A & Blundell TL (1993) Comparative protein modelling by satisfaction of spatial restraints. *J Mol Biol* 234**:** 779-815 http://salilab.org/modeller/
8. MacKerell Jr AD, Bashford D, Bellott M, Dunbrack Jr RL, Evanseck JD, Field MJ, Fischer S, Gao J, Guo H, Ha S, Joseph-McCarthy D, Kuchnir L, Kuczera K, Lau FTK, Mattos C, Michnick S, Ngo T, Nguyen DT, Prodhom B, Reiher III WE, Roux B, Schlenkrich M, Smith JC, Stote R, Straub J, Watanabe M, Wiorkiewicz-Kuczera J, Yin D, Karplus M (1998) All-atom empirical potential for molecular modeling and dynamics studies of proteins. *Journal of Physical Chemistry B* 102**:** 3586-3616
9. Baker NA, Sept D, Joseph S, Holst MJ, McCammon JA (2001), Electrostatics of nanosystems: application to microtubules and the ribosome. *Proc Natl Acad Sci USA* 98: 10037-10041 http://www.poissonboltzmann.org/apbs
10. Humphrey W, Dalke A and Schulten K (1996) VMD - Visual Molecular Dynamics. J. Molec. Graphics 14(1): 33-38 http://www.ks.uiuc.edu/Research/vmd/

Table S1. Strains and constructs used in this study

Strain/construct	Relevant genotype or description	Source
E. coli strains		
DH5α	*supE44ΔlacU169(ϕ80lacZΔM15)hsdR17 recA1 endA1 gyrA96 thi-1 relA1*	Lab stock
BW25113/ pIJ790	K12 derivative: Δ*araBAD*, Δ*rhaBAD* λ-Red*(gam,bet,exo), cat, araC, rep101*^{ts}	[1]
ET12567/ pUZ8002	*dam*-13::Tn*9,* dcm cat tet *hsd zjj-201::Tn10/tra neo* RP4	[2]
BTH101	F^-, *cya-99, araD139, galE15, galK16, rpsL1 (Str^r), hsdR2, mcrA1, mcrB1*	[3]
BL21 (DE3)pLysS		Lab stock
S. coelicolor strains		
M145	$SCP1^-$, $SCP2^-$	[2]
J2538	M145Δ*parAB*::*apra*	[4]
M145pK48	M145 pCJW93p$_{tipA}$*his-scy*	[5]
M145pK56	M145*attB*$_{\phi C31}$:: pIJ8660*p$_{scy}$-egfpscy*	[5]
K111	M145Δ*scy::apr*	[5]
DJ553	J3306pIJ6902(hygr)parA	[6]
BD08	M145*parA$_{Scy-}$*	This work
BD09	M145*parA$_{Scy-}$*, pIJ8660*p$_{scy}$ egfp-scy*	This work
BD10	M145*parA $_{Scy-}$*, pCJW93 p$_{tipA}$*his-scy*	This work
BD11	M145pCJW93	This work
BD12	M145*parA$_{Scy-}$*, pCJW93	This work
DJ595	M145*parA-egfp,* pK57(pIJ8660*p$_{scy}$ mchery-scy*)	This work
Constructs		
pUT18c	Vector carrying the T18 fragment for BTH	[3]
pKT25	Vector carrying the T25 fragment for BTH	[3]
pUT18parA	T18 translational fusion to ParA	[7]
pUT18CparAE250V	T18 translational fusion to ParA	This work
pUT18CparA $_{Scy-}$	T18 translational fusion to ParA $_{Scy-}$ (S249YE250V)	This work
pUT18c-Scy	T18 translational fusion to Scy	[8]
pKT25-Scy	T25 translational fusion to Scy	[8]
pUTc-ScyII	T18 translational fusion to ScyII	This work
pUTc-ScyIII	T18 translational fusion to ScyIII	This work
pUTc-ScyIV	T18 translational fusion to ScyIV	This work
pGEX6P2parA	pGEX6P2 derivative for the production of ParA	[7]
pGEX6P2parA$_{Scy-}$	pGEX6P2 derivative for the production of ParA$_{Scy-}$	This work
pGS2	pET28a derivative for the production of His-Scy	[5]
pIJ8660	plasmid integrating as a single copy at the ΦC31 *attB* attachment site on the chromosome of *S.coelicolor*	[9]
pCJW93	Multi-copy, autonomously replicating in *S.coelicolor*, carries thiostrepton-inducible promoter	[10]
pK48	A derivative of the multi-copy plasmid, pCJW93, carrying *scy* under the control of a thiostrepton-inducible promoter p$_{tipA}$*his-scy*	[5]
pK56	*egfp-scy* fusion under the control of native P*scy* in the integrative plasmid pIJ8660	[5]
pK57	*mcherry-scy* fusion under the control of native P*scy* in the integrative plasmid pIJ8660	[5]

References:

1. Gust B, Challis GL, Fowler K, Kieser T, Chater KF (2003) PCR-targeted *Streptomyces* gene replacement identifies a protein domain needed for biosynthesis of the sesquiterpene soil odor geosmin. *Proc Natl Acad Sci U S A* 100:1541-1546.
2. Kieser T, Bibb, M.J., Buttner, M.J., Chater, K.F., Hopwood, D.A. (2000) Practical *Streptomyces* Genetics. Norwich: The John Innes Foundation.
3. Karimova G, Pidoux J, Ullmann A, Ladant D (1998) A bacterial two-hybrid system based on a reconstituted signal transduction pathway. *Proc Natl Acad Sci U S A* 95:5752-5756.
4. Kim HJ, Calcutt MJ, Schmidt FJ, Chater KF (2000). Partitioning of the linear chromosome during sporulation of *Streptomyces coelicolor* A3(2) involves an *oriC*-linked *parAB* locus. *J Bacteriol.* 182, 1313-1320.
5. Holmes NA, Walshaw J, Thibessard A, Dalton KA, Leggett RM, Gillespie MD, Hemmings AM, Gust B, Kelemen GH. The assembly and maintenance of a bacterial polarity centre is organised by the novel scaffold protein, Scy. In press
6. Ditkowski B et al (2010) The actinobacterial signature protein ParJ (SCO1662) regulates ParA polymerization and affects chromosome segregation and cell division during *Streptomyces* sporulation. *Mol Microbiol* 78:1403-1415.
7. Jakimowicz D, Zydek P, Kois A, Zakrzewska-Czerwinska J, Chater KF (2007) Alignment of multiple chromosomes along helical ParA scaffolding in sporulating *Streptomyces* hyphae. *Mol Microbiol* 65(3):625-641.
8. Walshaw J, Gillespie MD, Kelemen GH (2010) A novel coiled-coil repeat variant in a class of bacterial cytoskeletal proteins. *J Struct Biol* 170: 202-215.
9. Sun, J., Kelemen, G.H., Fernandez-Abalos, J.M., and Bibb, M.J. (1999) Green fluorescent protein as a reporter for spatial and temporal gene expression in *Streptomyces coelicolor* A3(2). *Microbiology* 145, 2221-2227.
10. Wilkinson, CJ, Hughes-Thomas ZA, Martin CJ, Bohm I, Mironenko T, Deacon M, Wheatcroft M, Wirtz G, Staunton J, and Leadlay PF (2002) Increasing the efficiency of heterologous promoters in actinomycetes. *J Mol Microbiol Biotechnol* 4, 417-426.

Table S2. Primers used in the study

**Name**	Sequence	application
**Scy2965f**	CC**TCTAGAGGATCC**GCGGGCGCCCGGGCCGAG (*Xba*I, *Bam*HI)	*scyCIV* fragment into pUT18C i pKT25
**Scy_ENDR**	CC**GGTACCGAATTC**ATGCCGTCTGACGACTTGCCACC (*Kpn*I, *Eco*RI)	
**Scy3501R**	CC**GGTACCGAATTC**ATCTCGGAGGTGAGCCGCTCGC (*Kpn*I, *Eco*RI)	
**ScyIN_Fw**	CC**CTGCAG**G**AGATCT**GTGCGGGGCTACGAGAGCCAG (*Pst*I, *Bgl*II)	Cloning of *scyNhCI* into pUT18 i pUT18C
**ScyIN_Rv**	CC**TCTAGAGAATTC**ATCGCCTTGGACAGCTGGGTGGC (*Xba*I, *Eco*RI)	
**ScyIIN_F**	CC**TCTAGA**G**AGATCT**TCCGAGGCCGAGCAGGTGGTC (*Xba*I, *Bgl*II)	Cloning of *scyCII* into pUT18C i pKT25
**ScyIIN_R**	CC**GGTACCGAATTC**ATGGCGGCCTCCTCCTGGGCG (*Kpn*I, *Eco*RI)	
**ScyIIINF**	CC**TCTAGA**G**AGATCT**CGGCTGAAGACGGAGGCGCAG (*Xba*I, *Bgl*II)	Cloning of *scyCIII* into pUT18C i pKT25
**ScyIIINR**	CC**GGTACCGAATTC**ATCTCCTCGCGGGCCGCCGTG (*Kpn*I, *Eco*RI)	
**T18CseqF**	TACTTAGTTATATCGATG	Sequencing pUT18C insert
**T18CseqR**	CGTCGCTGGGCGCAGTGG	
**ETH_SA_F**	ACT CTA GAG ATG GAC GAC ACG CCG ATC GG	Amplification of *parA*
**ETH_SA_R**	GCGG TACCGC CTG GGT CCC CTC CAC CA	
**ParAB 4200**	CCGGATCCGGCTTCGCTCGGATGG	Verification of *parA*E250V mutation
**ParAEcorrv**	GC**GAATTC**ACTGGGTCCCCTCCACC (*Eco*RI)	
**YVmutfw**	CGGCGAGGAGGTGTTGCGGACGAGCATTCCCCGCTCGGTCC GTATC**TACGTA***ATTCCGGGGATCCGTCGAC*C (*Sna*BI)	Construction of p*arA*E250V mutation
**YVmutrv**	ACTTGATCCTGGATCGTAGGTCAGTACTGTCTGCCCGTAGCT CGGAGC**TACGTA***TGTAGGCTGGAGCTGCTTC* (*Sna*BI)	
**ParAgfp_fw**	AAGGGTGTGGGCGTCACCTATGACGCGACCCACGCCCACTTG GGG GCC CAA AAT GAC CCC TCG ATG GTC GAA GGC ACG CTGCCGGGCCCGGAGCTG	Construction *parA*-egfp strain
**A_gfp09R**	GGGGCGTTGGGGATCAGTGCACCGAGGCCACGACCCAACCC CCTCCGTCGCTCACTCATATGTGTAGGCTGGAGCTGCTTC	
**A_gfp_in**	TATGGAATTCGTGGAGGGGACCATATG	Insertion of oligonucleotide downstream of *parA-egfp*
**A_gfp_inR**	TATGGTCCCCTCCACGAATTCCA	
