# Supplementary material for: Dynamic interplay of ParA with the polarity protein, Scy, coordinates the growth with chromosome segregation in Streptomyces coelicolor
Source: Open Biol. 2013 Mar;3(3):130006. doi: 10.1098/rsob.130006 (PMC3718342; doi:10.1098/rsob.130006)
Supplement: Supporting Figures [file rsob130006-s2.pdf]

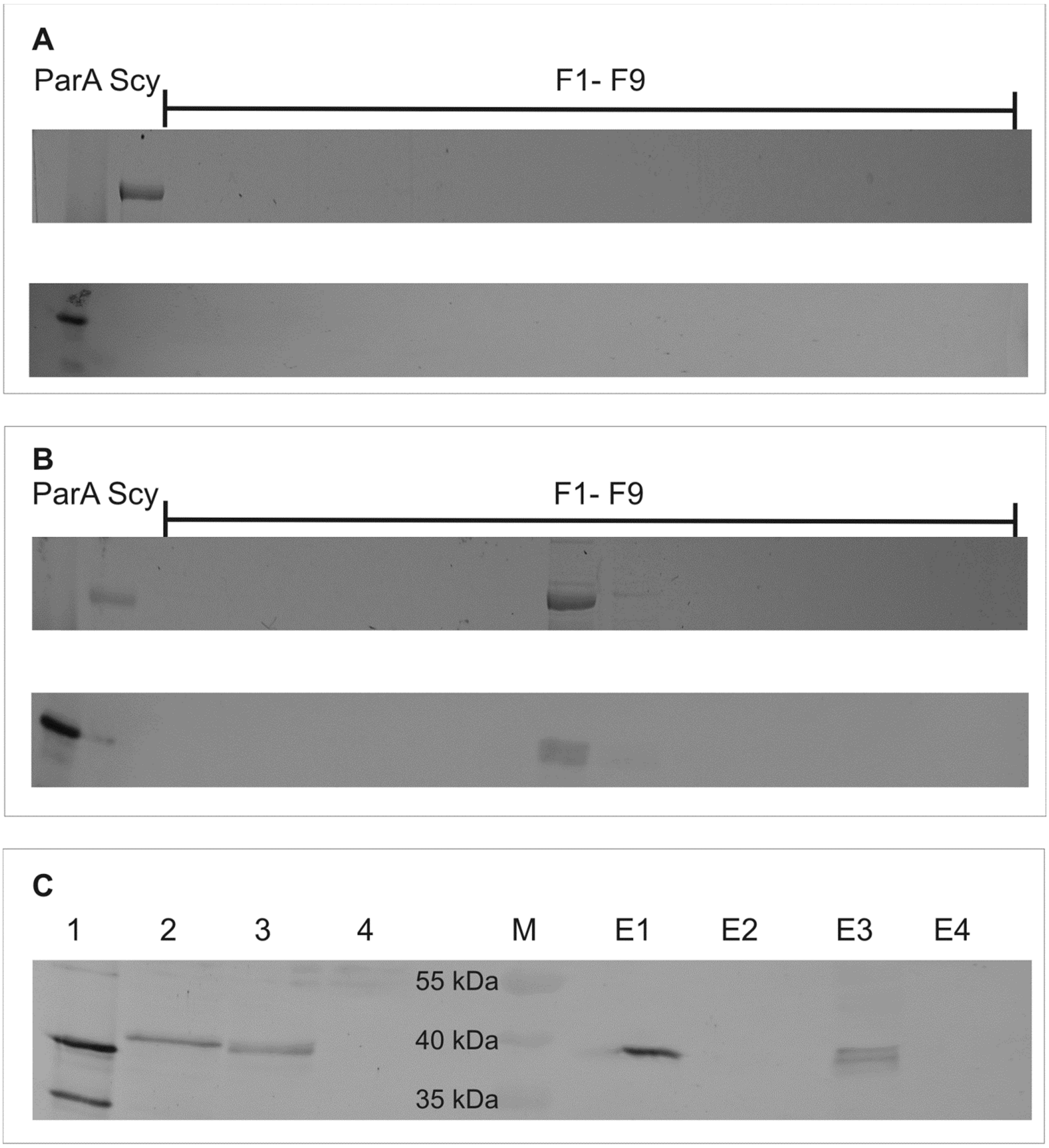


Fig. S1

**Fig. S1. Co-purification of 6His-Scy with ParA and ParA$_{Scy-}$ from *S. coelicolor* and the control experiments.**

A. The control negative experiment for ParA-Scy co-purification using metal-ion affinity chromatography performed with the extract of thiostrepton-induced strain BD11 (M145pCJW93) strain showing no unspecific interaction of ParA and Scy with the Ni-NA resin. Top panel SDS-PAGE, bottom panel Western-blotting with anti-ParA antibody.

B. Co-purification of $ParA_{Scy-}$ with 6His-Scy from the extract of thiostrepton-induced strain BD10 ($ParA_{Scy-}$, pCJW93$p_{tipA}$*his-scy*) using metal-ion affinity chromatography. Top panel SDS-PAGE, bottom panel Western-blotting with anti-ParA antibody.

C. Comparison of the efficiency of co-purification of ParA and $ParA_{Scy-}$ with 6His-Scy. Western-blotting with anti-ParA antibody of the selected elution fractions. The same amount of the protein was loaded from elution fraction of M145pK48 and from BD10. 1, purified ParA protein; 2, cell extract of M145pK48 (M145pCJW93$p_{tipA}$*his-scy*) strain; 3, cell extract of BD10 ($ParA_{Scy-}$, pCJW93$p_{tipA}$*his-scy*) strain 4, cell extract of J3306 (*ΔparA* strain); M, marker; E1, imidazole elution fraction of M145pK48 (M145pCJW93$p_{tipA}$*his-scy*) – positive result; E2, imidazole elution fractions of BD11 (M145pCJW93) – negative control; E3, imidazole elution fractions of BD10 ($ParA_{Scy-}$, pCJW93$p_{tipA}$*his-scy*) – weakened interaction; E4 imidazole elution fractions of BD12 ($ParA_{Scy-}$, pCJW93) – negative control.

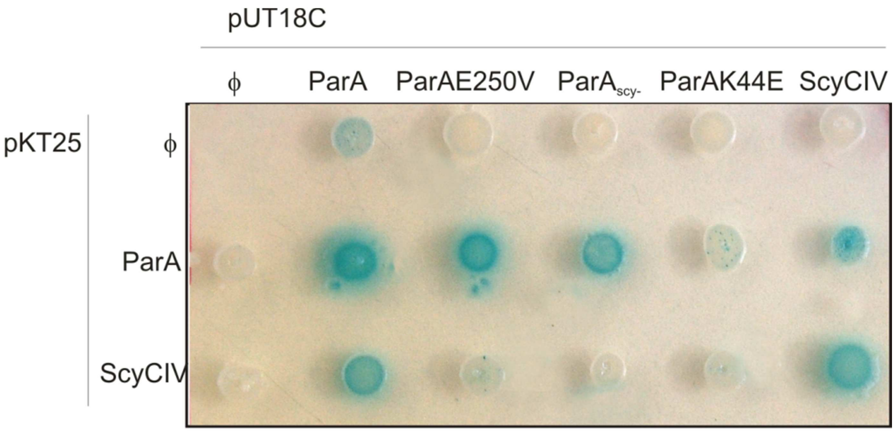


Fig. S2

**Fig. S2. Interaction of ParA mutants with Scy in the bacterial two hybrid system.**

Interaction of T18 fused to wild type ParA and mutant: ParAE250V (library), $ParA_{Scy-}$ (S249Y, E250V), ParAK44E with wild type ParA and ScyCIV fused with T25. Blue colony color indicates interaction of the proteins analyzed.

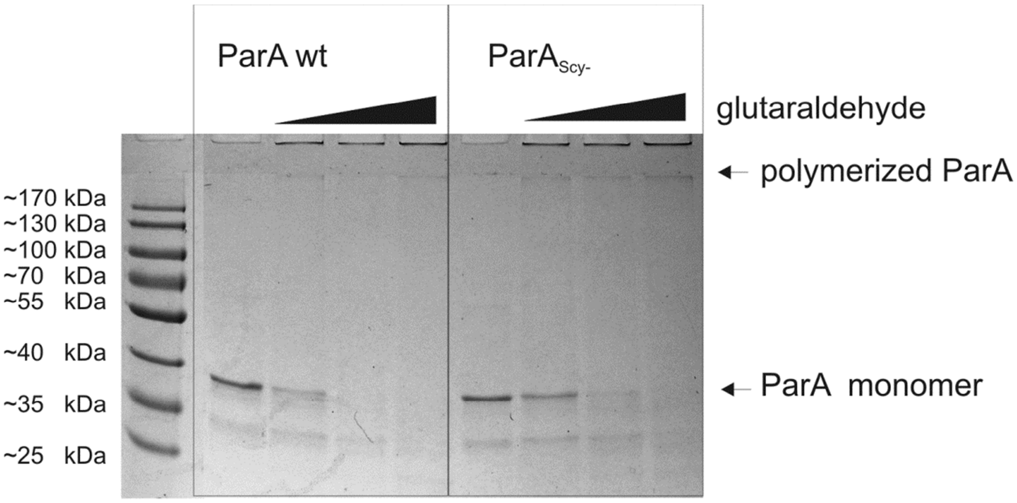


Fig. S3

**Fig. S3. Polymerization of the wild type ParA and ParA$_{scy-}$ assayed by glutaraldehyde crosslinking**. SDS-PAGE analysis of 5 μM ParA crosslinked with the increasing concentration of glutaraldehyde (2.5, 5 and 10 mM) in presence of 2 mM ATP

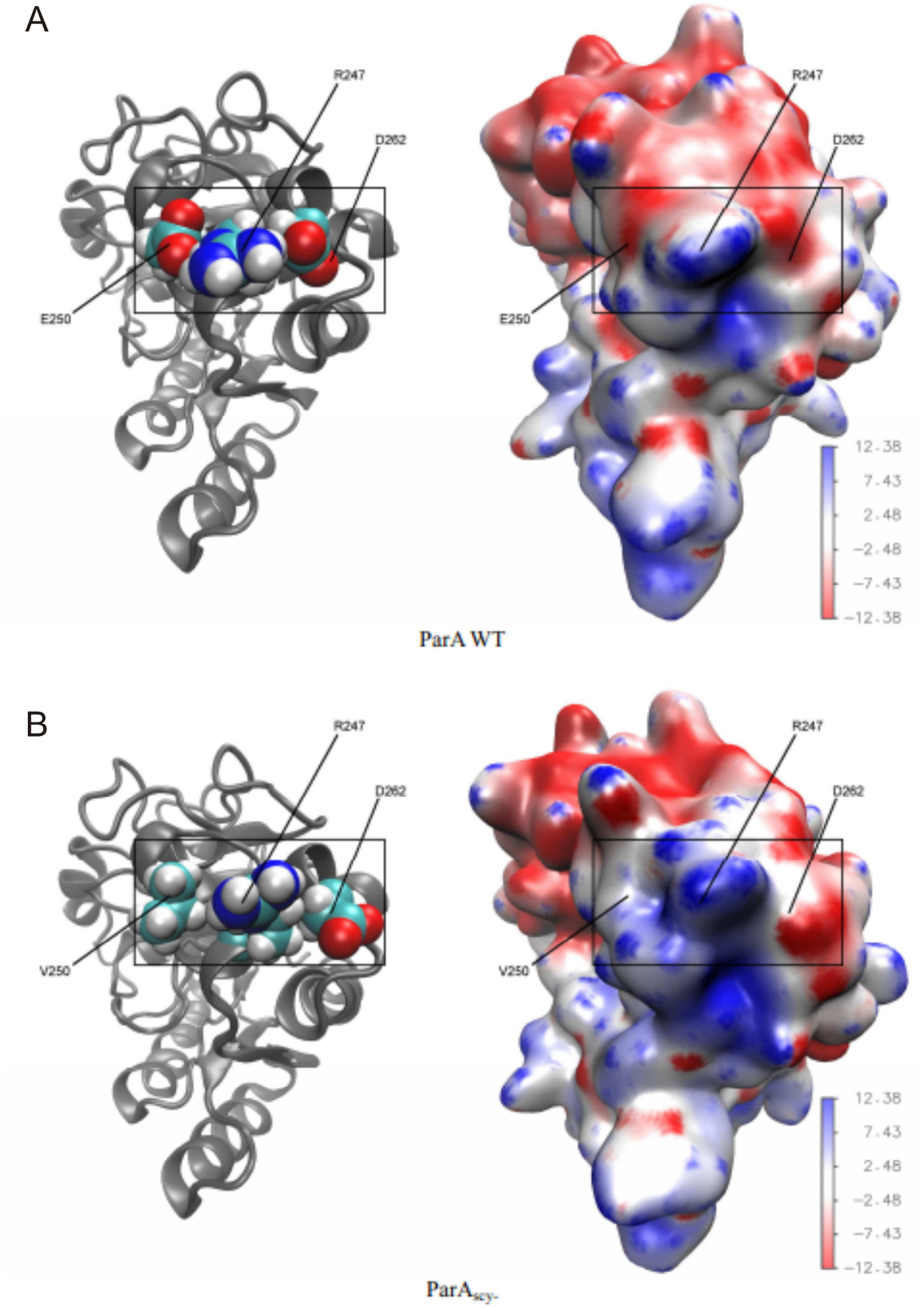


Fig. S4

**Fig. S4. The electrostatic potential on the surface of ParA (A) and ParA$_{scy-}$ (B) mapped using VMD.** Potential values in kJ/mol.

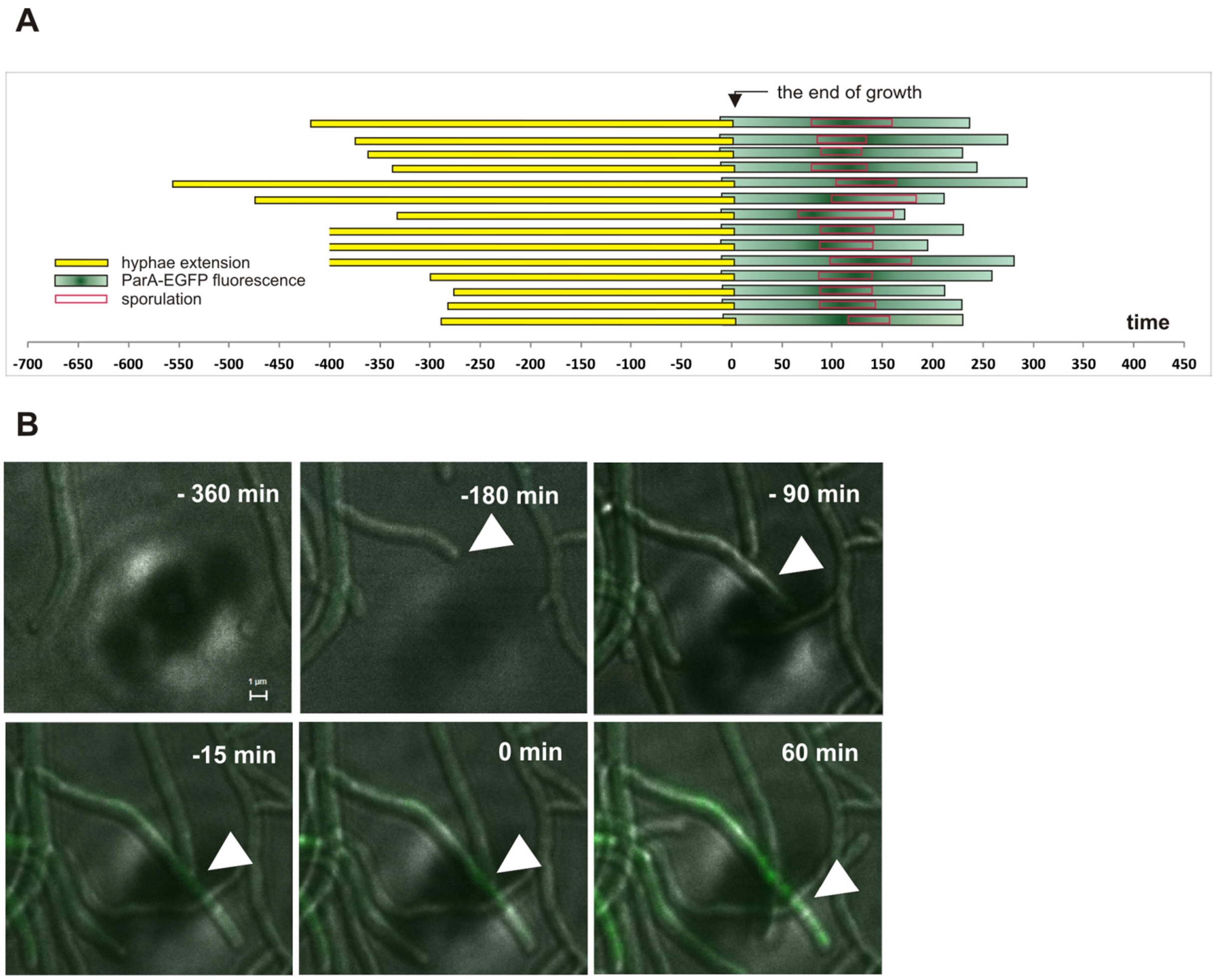


Fig. S5

**Fig. S5. Time lapse of *Streptomyces venezuelae* sporulating hyphae development showing close correlation of ParA-EGFP extension and the cessation of the hyphae growth.**
